# Supplementary material for: Metabolic syndrome and its associated factors among epileptic patients at Dessie Comprehensive Specialized Hospital, Northeast Ethiopia; a hospital-based comparative cross-sectional study
Source: PLoS One. 2022 Dec 29;17(12):e0279580. doi: 10.1371/journal.pone.0279580 (PMC9799290; doi:10.1371/journal.pone.0279580)
Supplement: S1 Fig — (DOCX) [file pone.0279580.s001.docx]

**Analysis on the authenticity of IDF criteria referring the NCEP-ATP III criteria**


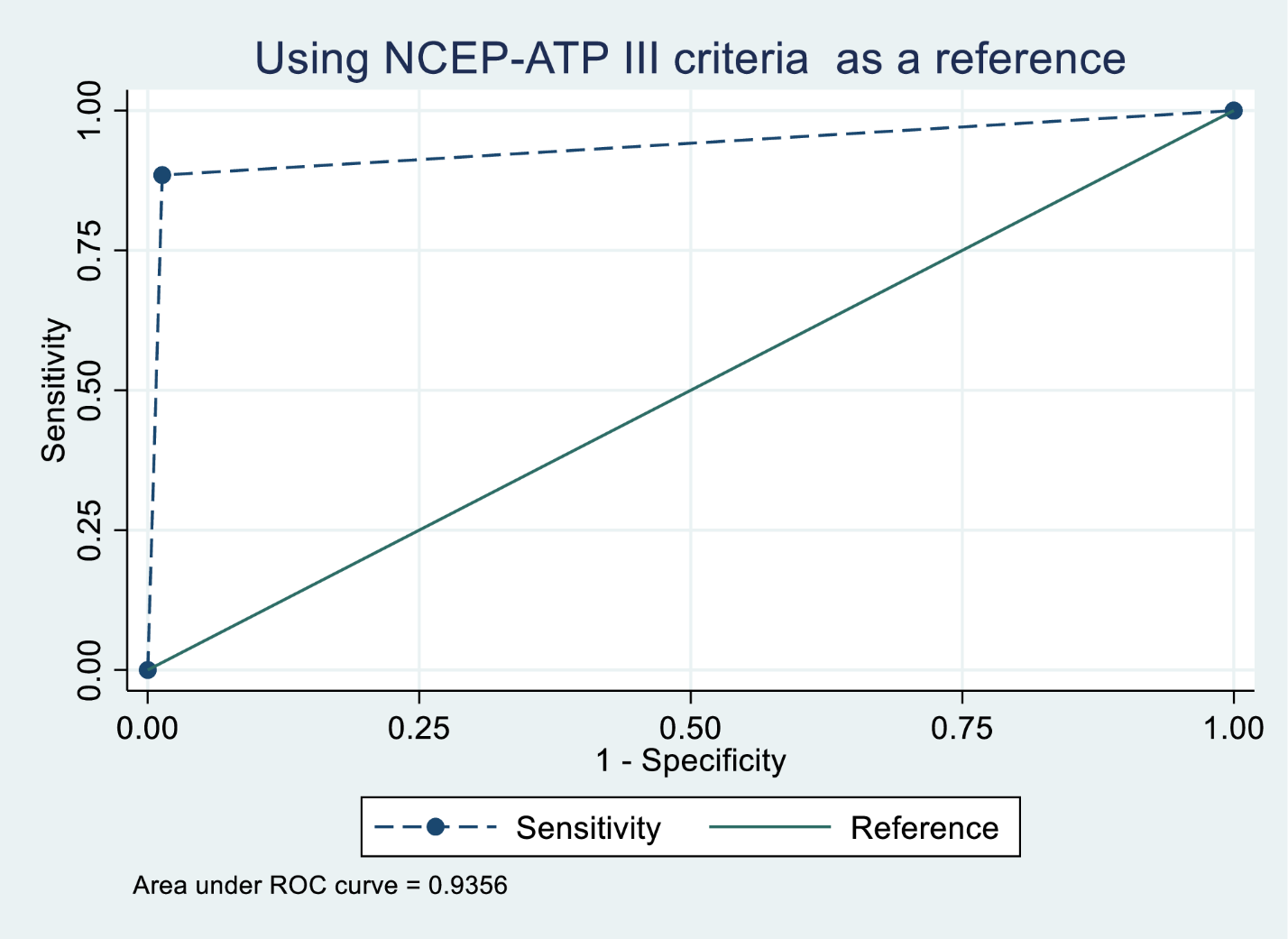


Fig 1. Receiver operating characteristic (ROC) curves of IDF criteria in epileptic group.

**Note**: Sensitivity = **88.46%, Specificity** = **98.67%**


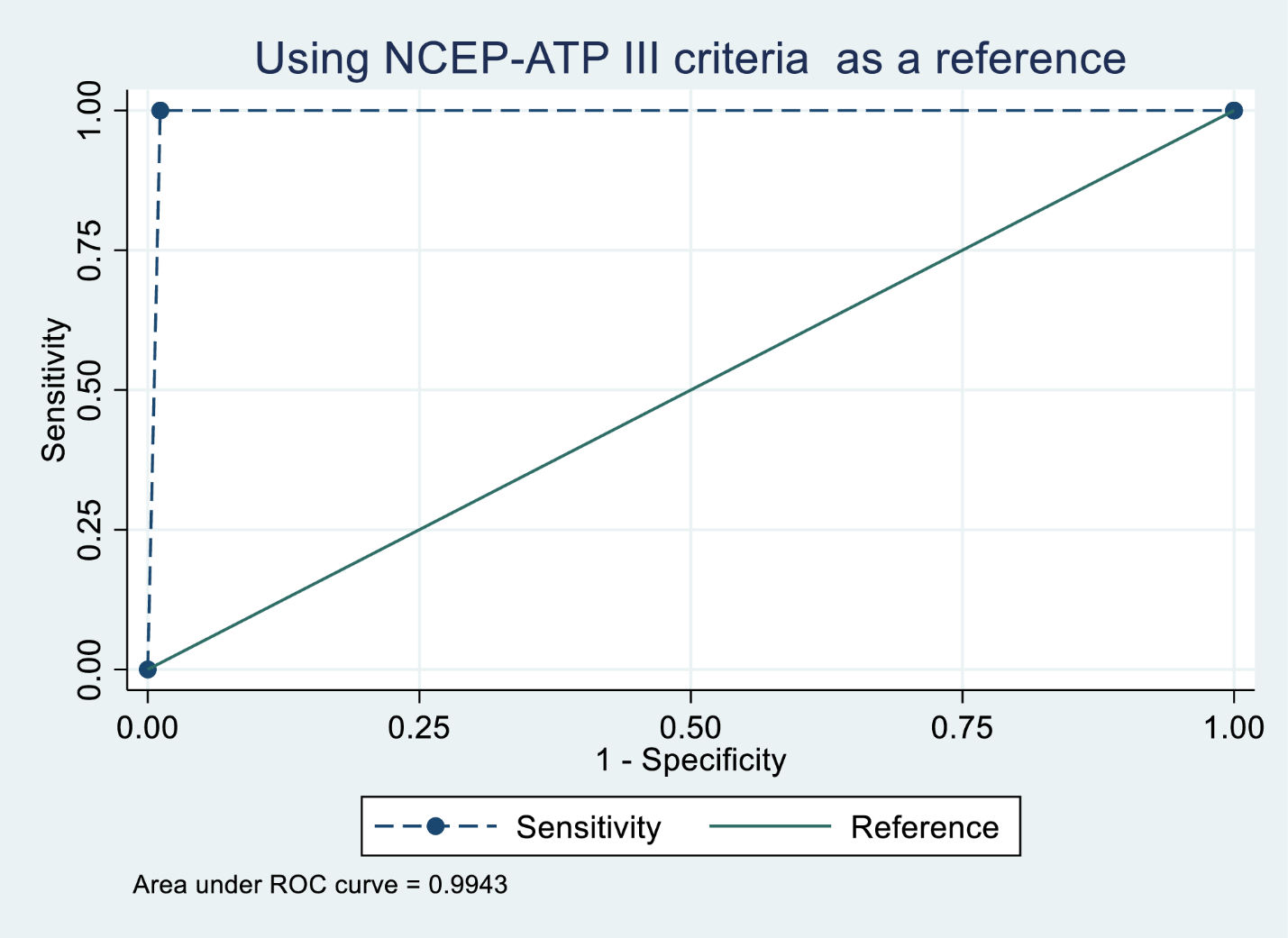


Fig 2. Receiver operating characteristic (ROC) curves of IDF criteria in control group

**Note:** Sensitivity = **100.00%, Specificity** = **98.85%**
