# Supplementary material for: Metabolic syndrome and its associated factors among epileptic patients at Dessie Comprehensive Specialized Hospital, Northeast Ethiopia; a hospital-based comparative cross-sectional study
Source: PLoS One. 2022 Dec 29;17(12):e0279580. doi: 10.1371/journal.pone.0279580 (PMC9799290; doi:10.1371/journal.pone.0279580)
Supplement: S1 File — (DOCX) [file pone.0279580.s002.docx]

**REVALENCE ASSESSMENT QUESTIONER**

WHO STEPS Instrument

Dessie, North East Ethiopia

## **English Version Questioner**

Dear respondents, given below are the items specifying necessary information expected from you for research. All your responses will be used in strict confidentiality in accomplishing the requirements of the study. Your honest answer to the questions in the questionnaire has an immense value to the completion of the study and you are kindly requested to give correct information accordingly. Thank you for your participation

## **Participant Identification Number ______**

##

Guide to the columns

| Column | Description | Site Tailoring |
| --- | --- | --- |
| Number | This question reference number is designed to help interviewers find their place if interrupted. | Renumber the instrument sequentially once the content has been finalized. |
| Question | Each question is to be read to the participants | - Select sections to use. - Add expanded and optional questions as desired. |
| Response | This column lists the available response options which the interviewer will be circling or filling in the text boxes. The skip instructions are shown on the right hand side of the responses and should be carefully followed during interviews. | - Change skips question identifiers from code to question number. |
| Code | The column is designed to match data from the instrument into the data entry tool, data analysis syntax, data book, and fact sheet. | This should never be changed or removed. The code is used as a general identifier for the data entry and analysis. |

| **Step 1 Demographic Information** |
| --- |

| **CORE: Demographic Information** | | | |
| --- | --- | --- | --- |
| **Question** | **Response** | | **Code** |
| Sex (*Record Male / Female as observed)* | Male | 1 | C1 |
|  | Female | 2 |  |
| How old are you? | Years | └─┴─┘ | C2 |

| **EXPANDED: Demographic Information** | | | |
| --- | --- | --- | --- |
| What is the **highest level of education** you have completed? | No formal schooling | 1 | C3 |
|  |  |  |  |
|  | Primary school completed | 2 |  |
|  | Secondary school completed | 3 |  |
|  | College/University completed | 4 |  |
|  | Post graduate degree | 5 |  |
|  | Refused | 88 |  |
| Which of the following best describes your **main** **work** status over the past 12 months? | Government employee | 1 | C5 |
|  | Non-government Employee | 2 |  |
|  | Private employee | 3 |  |
|  | Farmer | 4 |  |
|  | Trader | 5 |  |
|  | Retired | 6 |  |
|  | Daily labor | 7 |  |
|  | Others | 8 __________________________ |  |
|  | Refused | 88 |  |

| **Question** | **Response** | | | **Code** |
| --- | --- | --- | --- | --- |
| Taking **the past year**, can you tell me what the average earnings (Birr) of the household have been?  *(RECORD ONLY ONE, NOT ALL 3)* | Per week | └─┴─┴─┴─┴─┴─┴─┘ *Go to T1* | | C6a |
|  | OR per month | └─┴─┴─┴─┴─┴─┴─┘  *Go to T1* | | C6b |
|  | OR per year | └─┴─┴─┴─┴─┴─┴─┘ *Go to T1* | | C6c |
|  | Refused | 88 | | C6d |
| If you don’t know the amount, can you give an **estimate** of the annual household income if I read some options to you? Is it  *(READ OPTIONS)* | ≤ 12,000 Birr | | 1 | C7 |
|  | More than 12,000 ≤ 18,000 Birr | | 2 |  |
|  | More than 18,000 ≤23,300 | | 3 |  |
|  | More than 23,300 ≤ 30,000 | | 4 |  |
|  | More than 30,000 | | 5 |  |
|  | Don't Know | | 77 |  |
|  | Refused | | 88 |  |

| **Step 2 Behavioural Measurements** |
| --- |

| **CORE: Tobacco Use** | | | |
| --- | --- | --- | --- |
| Now I am going to ask you some questions about tobacco use. | | | |
| **Question** | **Response** | | **Code** |
| Do you **currently** smoke any **tobacco** products, such as cigarettes, cigars or pipes, *gaya*?  *(USE SHOWCARD)* | Yes | 1 | T1 |
|  | No | 2 *If No, go to T8* |  |
| Do you currently smoke tobacco products **daily**? | Yes | 1 | T2 |
|  | No | 2 |  |
| How old were you when you **first started** smoking? | Age (years) | **└─┴─┘** *If Known, go to T5a/T5aw* | T3 |
|  | Don’t know 77 |  |  |
| Do you remember how long ago it was?  *(RECORD ONLY 1, NOT ALL 3)*  *Don’t know 77* | In Years | **└─┴─┘** *If Known, go to T5a/T5aw* | T4a |
|  | OR in Months | **└─┴─┘** *If Known, go to T5a/T5aw* | T4b |
|  | OR in Weeks | **└─┴─┘** | T4c |
| On average, **how many** of the following products do you smoke **each day/week?**  *(IF LESS THAN DAILY, RECORD WEEKLY)*  *(RECORD FOR EACH TYPE, USE SHOWCARD)*  *Don’t Know 7777* | DAILY**↓** WEEKLY↓ | | |
|  | Manufactured cigarettes | └─┴─┴─┴**─┘**└─┴─┴─┴**─┘** | T5a/T5aw |
|  | Hand-rolled cigarettes | └─┴─┴─┴**─┘**└─┴─┴─┴**─┘** | T5b/T5bw |
|  | Pipes full of tobacco | └─┴─┴─┴**─┘**└─┴─┴─┴**─┘** | T5c/T5cw |
|  | Number of Shisha sessions | └─┴─┴─┴**─┘**└─┴─┴─┴**─┘** | T5e/T5ew |
|  | Gaya | └─┴─┴─┴**─┘**└─┴─┴─┴**─┘** | T5X/  T5Xw |
|  | Other | └─┴─┴─┴**─┘**└─┴─┴─┴**─┘** *If Other, go to T5other, else go to T6* | T5f/T5fw |
|  | Other (please specify): | ______________________________ | T5other/  T5otherw |
| During the past 12 months, have you tried to **stop smoking**? | Yes | 1 | T6 |
|  | No | 2 |  |
| During any visit to a doctor or other health worker in the past 12 months, were you advised to quit smoking tobacco? | Yes | 1 *If T2=Yes, go to T12; if T2=No, go* *to T9* | T7 |
|  | No | 2 *If T2=Yes, go to T12; if T2=No, go to T9* |  |
|  | No visit during the past 12 months | 3 *If T2=Yes, go to T12; if T2=No, go to T9* |  |
| In the past, did you **ever** **smoke** any tobacco products?  *(USE SHOWCARD)* | Yes | 1 | T8 |
|  | No | 2 *If No, go to T12* |  |
| In the past, did you **ever** smoke **daily**? | Yes | 1 *If T1=Yes, go to T12, else go to T10* | T9 |
|  | No | 2 *If T1=Yes, go to T12, else go to T10* |  |

| How old were you when you **stopped** smoking? | Age (years) | **└─┴─┘** *If Known, go to T12* | T10 |
| --- | --- | --- | --- |
|  | Don’t Know 77 |  |  |
| How **long ago** did you stop smoking?  *(RECORD ONLY 1, NOT ALL 3)*  *Don’t Know 77* | Years ago | **└─┴─┘** *If Known, go to T12* | T11a |
|  | OR Months ago | **└─┴─┘** *If Known, go to T12* | T11b |
|  | OR Weeks ago | **└─┴─┘** | T11c |

| **CORE: Tobacco Use, cont.** | | | | |
| --- | --- | --- | --- | --- |
| **Question** | **Response** | | | **Code** |
| Do you **currently use** any **smokeless tobacco** products such as *snuff(Suret), chewing tobacco, (USE SHOWCARD)* | | Yes | 1 | T12 |
|  |  | No | 2 *If No, go to T15* |  |
| Do you **currently use** **smokeless tobacco** products **daily?** | | Yes | 1 | T13 |
|  |  | No | 2 *If No, go to T14aw* |  |
| On average, how many **times a day/week** do you use ….  *(IF LESS THAN DAILY, RECORD WEEKLY)*  *(RECORD FOR EACH TYPE, USE SHOWCARD)*  *Don’t Know 7777* | | DAILY**↓** WEEKLY↓ | | |
|  |  | Snuff, by mouth | └─┴─┴─┴**─┘**└─┴─┴─┴**─┘** | T14a/  T14aw |
|  |  | Snuff, by nose | └─┴─┴─┴**─┘**└─┴─┴─┴**─┘** | T14b/  T14bw |
|  |  | Chewing tobacco | └─┴─┴─┴**─┘**└─┴─┴─┴**─┘** | T14c/  T14cw |
|  |  | Other | └─┴─┴─┴**─┘**└─┴─┴─┴**─┘** *If Other, go to T14other, if T13=No, go to T16, else go to T17* | T14e/  T14ew |
|  |  | Other (please specify): | *_______________________________If T13=No, go to T16, else go to T17* | T14other/  T14otherw |
| In the **past**, did you **ever use** smokeless tobacco products such as *snuff, chewing tobacco*? | | Yes | 1 | T15 |
|  |  | No | 2 *If No, go to T17* |  |
| In the **past**, did you **ever use** smokeless tobacco products such as *snuff, chewing tobacco daily*? | | Yes | 1 | T16 |
|  |  | No | 2 |  |
| During the past 30 days, did someone smoke **in your home**? | | Yes | 1 | T17 |
|  |  | No | 2 |  |
| During the past 30 days, did someone smoke in closed areas **in your workplace** (in the building, in a work area or a specific office)? | | Yes | 1 | T18 |
|  |  | No | 2 |  |
|  |  | Don't work in a closed area | 3 |  |

| **CORE: Alcohol Consumption** | | | |
| --- | --- | --- | --- |
| The next questions ask about the consumption of alcohol. | | | |
| **Question** | **Response** | | **Code** |
| Have you **ever** consumed any alcohol such beer, Tella, Bordie, Tej, Arake, wine, spirits, beherawi, ye bale zaf?  *(USE SHOWCARD OR SHOW EXAMPLES)* | Yes | 1 | A1 |
|  | No | 2  *If No, go to A16* |  |
| Have you consumed any alcohol within the **past 12 months**? | Yes | 1  *If Yes, go to A4* | A2 |
|  | No | 2 |  |
| Have you stopped drinking due to health reasons, such as a negative impact on your health or on the advice of your doctor or other health worker? | Yes | 1 *If Yes, go to A16* | A3 |
|  | No | 2 *If No, go to A16* |  |
| During the past 12 months, **how frequently** have you had at least one standard alcoholic drink?  *(READ RESPONSES, USE SHOWCARD)* | Daily | 1 | A4 |
|  | 5-6 days per week | 2 |  |
|  | 3-4 days per week | 3 |  |
|  | 1-2 days per week | 4 |  |
|  | 1-3 days per month | 5 |  |
|  | Less than once a month | 6 |  |
| Have you consumed any alcohol within the **past 30 days**? | Yes | 1 | A5 |
|  | No | 2 *If No, go to A13* |  |
| During the past 30 days, on how many **occasions** did you have at least one standard alcoholic drink? | Number  Don't know 77 | └─┴─┘ | A6 |
| During the past 30 days, when you drank alcohol, how many **standard** **drinks on average** did you have during one drinking occasion?  *(USE SHOWCARD)* | Number  Don't know 77 | └─┴─┘ | A7 |
| During the past 30 days, what was the **largest number** of standard drinks you had on a single occasion, counting all types of alcoholic drinks together? | Largest number  Don't Know 77 | └─┴─┘ | A8 |
| During the past 30 days, how many times did you have  **six or more** standard drinks in a single drinking occasion? | Number of times Don't Know 77 | └─┴─┘ | A9 |
| During the past 30 days, when you consumed an alcoholic drink, how often was it with meals? Please do not count snacks. | Usually with meals 1  Sometimes with meals 2  Rarely with meals 3  Never with meals 4 | | X6 |
| During each of the **past 7 days**, how many standard drinks did you have each day?  *(USE SHOWCARD)*  *Don't Know 77* | Monday | └─┴─┘ | A10a |
|  | Tuesday | └─┴─┘ | A10b |
|  | Wednesday | └─┴─┘ | A10c |
|  | Thursday | └─┴─┘ | A10d |
|  | Friday | └─┴─┘ | A10e |
|  | Saturday | └─┴─┘ | A10f |
|  | Sunday | └─┴─┘ | A10g |

| **CORE: Alcohol Consumption, cont.** | | | |
| --- | --- | --- | --- |
| I have just asked you about your consumption of alcohol during the past 7 days. The questions were about alcohol in general, while the next questions refer to your consumption of homebrewed alcohol, alcohol brought over the border/from another country, any alcohol not intended for drinking or other untaxed alcohol. Please only think about these types of alcohol when answering the next questions. | | | |
| **Question** | **Response** | | **Code** |
| During the **past 7 days**, did you consume any **homebrewed** alcohol, like Tella, Tej, Katikalla, Bordie?  *(USE SHOWCARD)* | Yes | 1 | A11 |
|  | No | 2 *If No, go to A13* |  |
| On average, **how many standard drinks** of the following did you consume **during the past 7 days**?  *(USE SHOWCARD)*  *Don't Know 77* | Homebrewed spirits, e.g. Katikala | **└─┴─┘** | A12a |
|  | Homebrewed beer or wine, e.g. Tella, Tej | **└─┴─┘** | A12b |
|  | Alcohol brought over the border/from another country | **└─┴─┘** | A12c |
|  | Alcohol not intended for drinking, e.g. alcohol-based medicines, perfumes, after shaves | **└─┴─┘** | A12d |
|  | Other untaxed alcohol in the country | **└─┴─┘** | A12e |
| During the **past 12 months,** how often have you found that you were not able to stop drinking once you had started? | Daily or almost daily | 1 | A13 |
|  | Weekly | 2 |  |
|  | Monthly | 3 |  |
|  | Less than monthly | 4 |  |
|  | Never | 5 |  |
| During the **past 12 months,** how often have you failed to do what was normally expected from you because of drinking? | Daily or almost daily | 1 | A14 |
|  | Weekly | 2 |  |
|  | Monthly | 3 |  |
|  | Less than monthly | 4 |  |
|  | Never | 5 |  |
| During the **past 12 months,** how often have you needed a first drink in the morning to get yourself going after a heavy drinking session? | Daily or almost daily | 1 | A15 |
|  | Weekly | 2 |  |
|  | Monthly | 3 |  |
|  | Less than monthly | 4 |  |
|  | Never | 5 |  |
| During the **past 12 months**, have you had family problems or problems with your partner due to **someone else’s** drinking? | Yes, more than monthly | 1 | A16 |
|  | Yes, monthly | 2 |  |
|  | Yes, several times but less than monthly | 3 |  |
|  | Yes, once or twice | 4 |  |
|  | No | 5 |  |

| **Khat use** | | | | | | | |
| --- | --- | --- | --- | --- | --- | --- | --- |
| Now I am going to ask you some questions about Khat chewing. | | | | | | | |
| **Question** | | | **Response** | | | **Code** | |
| Have you ever chewed Khat?  *(USE SHOWCARD)* | | | Yes | 1 | | K1 | |
|  |  |  | No | 2 *If No, go to K14* | |  |  |
| Do you currently chew Khat? | | | Yes | 1 | | K2 | |
|  |  |  | No | 2 If No, go to K8 | |  |  |
| During the past 12 months, how frequently did you chew Khat? | | | Daily  5-6 days per week  3-4 days per week  1-2 days per week  1-3 days per month  Less than once a month | 1  2  3  4  5  6 | | K3 | |
| How old were you when you **first started** chewing Khat? | | | Age (years) **└─┴─┘** | *If Known, go to K5* | | K4 | |
|  |  |  | Don’t know 77 |  |  |  |  |
| Do you remember how long ago it was?  *(RECORD ONLY 1, NOT ALL 3)*  *Don’t know 77* | | | In Years | **└─┴─┘** *If Known, go to K5* | | K4a | |
|  |  |  | OR in Months | **└─┴─┘** *If Known, go to K5* | | K4b | |
|  |  |  | OR in Weeks | **└─┴─┘** | | K4c | |
| On average, **how many** bundles of Khat do you chew **each day/week?**  *(IF LESS THAN DAILY, RECORD WEEKLY)*  *(USE SHOWCARD)*  *Don’t Know 77* | | | DAILY**↓** WEEKLY↓ | | | | |
|  |  |  | Bundles of Khat | └─┴**─┘** └─┴**─┘** | | K5 | |
| During the past 12 months, have you tried to **stop chewing Khat**? | | | Yes | 1 | | K6 | |
|  |  |  | No | 2 | |  |  |
| During any visit to a doctor or other health worker in the past 12 months, were you advised to **quit chewing Khat**? | | | Yes | 1 go to K9 | | K7 | |
|  |  |  | No | 2 *go* *to K9* | |  |  |
|  |  |  | No visit during the past 12 months | 3 *go* *to K9* | |  |  |
| How old were you when you **stopped** chewing Khat? | Age (years) | | | **└─┴─┘** *If Known, go to K10* | | K8 | |
| How long ago did you stop chewing Khat?  (RECORD ONLY 1, NOT ALL 3)  Don’t Know 77 | Years ago | | | **└─┴─┘ If Known, go to K10** | | K8a | |
|  | OR Months ago | | | **└─┴─┘ If Known, go to K10** | | K8b | |
|  | OR Weeks ago | | | **└─┴─┘if known, go to K10** | | K8c | |
| Do you currently smoke tobacco products while chewing Khat?  (USE TOBACCO SHOWCARD) | Yes  No | | | 1 If yes, go to K11  2 | | K9 | |
| In the **past**, did you **ever smoke** tobacco products while chewing Khat? | Yes  No | | | 1 If K2=2 go to K13  2 If K2=2 go to K13 | | K10 | |
| Does one or more of your friends smoke tobacco products while you chew Khat together? | Yes  No | | | 1  2 | | K11 | |
| Do you currently drink alcohol after you chew Khat? | Yes  No | | | 1 If yes, go to K14 | | K12 | |
| In the **past**, did you **ever drink alcohol** while chewing Khat? | Yes  No | | | 1  2 | | K13 | |
| During the past 12 months, have you had family problems or problems with your partner due to consumption of Khat by you or somebody else? | Yes  No | | | 1  2 | | K14 | |

| **Diet** | | | |
| --- | --- | --- | --- |
| The next questions ask about the fruits and vegetables that you usually eat. I have a nutrition card here that shows you some examples of local fruits and vegetables. Each picture represents the size of a serving. As you answer these questions please think of a typical week in the last year. | | | |
| **Question** | **Response** | | **Code** |
| In a typical week, on how many days do you **eat fruit**?  *(USE SHOWCARD)* | Number of days Don't Know 77 | └─┴─┘ If Zero days, go to D3 | D1 |
| How many **servings** of fruit do you eat on **one** of those days? (*USE SHOWCARD)* | Number of servings  Don't Know 77 | └─┴─┘ | D2 |
| In a typical week, on how many days do you **eat vegetables**? *(USE SHOWCARD)* | Number of days Don't Know 77 | └─┴─┘ If Zero days, go to D5 | D3 |
| How many **servings** of vegetables do you eat on one of those days? *(USE SHOWCARD)* | Number of servings  Don’t know 77 | └─┴─┘ | D4 |

| **CORE: Physical Activity** | | | |
| --- | --- | --- | --- |
| Next I am going to ask you about the time you spend doing different types of physical activity in a typical week. Please answer these questions even if you do not consider yourself to be a physically active person.  Think first about the time you spend doing work. Think of work as the things that you have to do such as paid or unpaid work, study/training, household chores, harvesting food/crops, fishing or hunting for food, seeking employment. In answering the following questions 'vigorous-intensity activities' are activities that require hard physical effort and cause large increases in breathing or heart rate, 'moderate-intensity activities' are activities that require moderate physical effort and cause small increases in breathing or heart rate. | | | |
| **Question** | **Response** | | **Code** |
| **Work** | | | |
| Does your work involve vigorous-intensity activity that causes large increases in breathing or heart rate like *carrying or lifting* *heavy loads, digging or construction work, cutting fire and other wood* for at least 10 minutes continuously?  *(USE SHOWCARD)* | Yes | 1 | P1 |
|  | No | 2  *If No, go to P 4* |  |
| In a typical week, on how many days do you do vigorous-intensity activities as part of your work? | Number of days | └─┘ | P2 |
| How much time do you spend doing vigorous-intensity activities at work on a typical day? | Hours : minutes | └─┴─┘: └─┴─┘  hrs mins | P3 (a-b) |
| Does your work involve/ or do you do moderate-intensity activity, that causes small increases in breathing or heart rate such as brisk walking *or carrying light loads, washing clothes* for at least 10 minutes continuously?  *(USE SHOWCARD)* | Yes | 1 | P4 |
|  | No | 2 *If No, go to P 7* |  |
| In a typical week, on how many days do you do moderate-intensity activities as part of your work? | Number of days | └─┘ | P5 |
| How much time do you spend doing moderate-intensity activities at work on a typical day? | Hours : minutes | └─┴─┘: └─┴─┘  hrs mins | P6 (a-b) |
| **Travel to and from places** | | | |
| The next questions exclude the physical activities at work that you have already mentioned.  Now I would like to ask you about the usual way you travel to and from places. For example to work, for shopping, to market, to place of worship, to place of meeting. | | | |
| Do you walk or use a bicycle *(pedal cycle)* for at least 10 minutes continuously to get to and from places? | Yes | 1 | P7 |
|  | No | 2  *If No, go to P 10* |  |
| In a typical week, on how many days do you walk or bicycle for at least 10 minutes continuously to get to and from places? | Number of days | └─┘ | P8 |
| How much time do you spend walking or bicycling for travel on a typical day? | Hours : minutes | └─┴─┘: └─┴─┘  hrs mins | P9 (a-b) |

| **Recreational activities** | | | |
| --- | --- | --- | --- |
| The next questions exclude the work and transport activities that you have already mentioned.  Now I would like to ask you about sports, fitness and recreational/leisure activities. | | | |
| Do you do any vigorous-intensity sports, fitness or recreational *(leisure)* activities that cause large increases in breathing or heart rate like *running or football, local dancing* for at least 10 minutes continuously?  *(USE SHOWCARD)* | Yes | 1 | P10 |
|  | No | 2  *If No, go to P 13* |  |
| In a typical week, on how many days do you do vigorous-intensity sports, fitness or recreational *(leisure)* activities? | Number of days | └─┘ | P11 |
| How much time do you spend doing vigorous-intensity sports, fitness or recreational activities on a typical day? | Hours : minutes | └─┴─┘: └─┴─┘  hrs mins | P12  (a-b) |

| **Physical Activity, Continued** | | | |
| --- | --- | --- | --- |
| **Question** | **Response** | | **Code** |
| Do you do any moderate-intensity sports, fitness or recreational (leisure) activities that cause a small increase in breathing or heart rate such as brisk walking, cycling, swimming, volleyball for at least 10 minutes continuously?  (USE SHOWCARD) | Yes | 1 | P13 |
|  | No | 2  *If No, go to P16* |  |
| In a typical week, on how many days do you do moderate-intensity sports, fitness or recreational (leisure) activities? | Number of days | └─┘ | P14 |
| How much time do you spend doing moderate-intensity sports, fitness or recreational *(leisure)* activities on a typical day? | Hours : minutes | └─┴─┘: └─┴─┘  hrs mins | P15 (a-b) |

| **Physical Activity** | | | |
| --- | --- | --- | --- |
| **Sedentary behavior** | | | |
| The following question is about sitting or reclining at work, at home, getting to and from places, or with friends including time spent sitting at a desk, sitting with friends, traveling in car, bus, train, reading, playing games/cards or watching television, but do not include time spent sleeping.  *(USE SHOWCARD)* | | | |
| How much time do you usually spend sitting or reclining on a typical day? | Hours : minutes | └─┴─┘: └─┴─┘  hrs mins | P16  (a-b) |

| **History of Raised Blood Pressure** | | | |
| --- | --- | --- | --- |
| **Question** | **Response** | | **Code** |
| Have you ever had your blood pressure measured by a doctor or other health worker? | Yes | 1 | H1 |
|  | No | 2 *If No, go to X10* |  |
| Have you ever been told by a doctor or other health worker that you have raised blood pressure or hypertension? | Yes | 1 | H2a |
|  | No | 2  *If No, go to X10* |  |
| Have you been told in the past 12 months? | Yes | 1 | H2b |
|  | No | 2 |  |
| In the past two weeks, have you taken any drugs (medication) for raised blood pressure prescribed by a doctor or other health worker? | Yes | 1 | H3 |
|  | No | 2 |  |
| Have you ever seen a traditional healer for raised blood pressure or hypertension? | Yes | 1 | H4 |
|  | No | 2 |  |
| Are you currently taking any herbal or traditional remedy for your raised blood pressure? | Yes | 1 | H5 |
|  | No | 2 |  |
| Has any of your family members (biological parents, siblings or children) ever had raised blood pressure or hypertension? | Yes | 1 | X10 |
|  | No | 2 |  |

| **History of Diabetes** | | | |
| --- | --- | --- | --- |
| Have you ever had your blood sugar measured by a doctor or other health worker? | Yes | 1 | H6 |
|  | No | 2 *If No, go to X11* |  |
| Have you ever been told by a doctor or other health worker that you have raised blood sugar or diabetes? | Yes | 1 | H7a |
|  | No | 2  *If No, go to X11* |  |
| Have you been told in the past 12 months? | Yes | 1 | H7b |
|  | No | 2 |  |
| In the past two weeks, have you taken any drugs (medication) for diabetes prescribed by a doctor or other health worker? | Yes | 1 | H8 |
|  | No | 2 |  |
| Are you currently taking insulin for diabetes prescribed by a doctor or other health worker? | Yes | 1 | H9 |
|  | No | 2 |  |
| Have you ever seen a traditional healer for diabetes or raised blood sugar? | Yes | 1 | H10 |
|  | No | 2 |  |
| Are you currently taking any herbal or traditional remedy for your diabetes? | Yes | 1 | H11 |
|  | No | 2 |  |
| Has any of your family members (biological parents, siblings or children) ever had raised blood sugar or Diabetes? |  | Yes 1  No 2 | X11 |

| **History of Raised Total Cholesterol** | | | | | | | |
| --- | --- | --- | --- | --- | --- | --- | --- |
| **Question** | **Response** | | | | **Code** | |  |
| Have you ever had your cholesterol (fat levels in your blood) measured by a doctor or other health worker? | | | Yes | 1 | H12 | |  |
|  |  |  | No | 2 *If No, go to X12* |  |  |  |
| Have you ever been told by a doctor or other health worker that you have raised cholesterol? | | | Yes | 1 | H13a | |  |
|  |  |  | No | 2 *If No, go to X12* |  |  |  |
| Have you been told in the past 12 months? | | | Yes | 1 | H13b | |  |
|  |  |  | No | 2 |  |  |  |
| In the past two weeks, have you taken any oral treatment (medication) for raised total cholesterol prescribed by a doctor or other health worker? | | | Yes | 1 | H14 | |  |
|  |  |  | No | 2 |  |  |  |
| Have you ever seen a traditional healer for raised cholesterol? | | | Yes | 1 | H15 | |  |
|  |  |  | No | 2 |  |  |  |
| Are you currently taking any herbal or traditional remedy for your raised cholesterol? | | | Yes | 1 | H16 | |  |
|  |  |  | No | 2 |  |  |  |
| Has any of your family members (biological parents, siblings or children) ever had raised Cholesterol? | | | Yes | 1 | X12 | |  |
|  |  |  | No | 2 |  |  |  |

**Clinical characteristics and therapy of epilepsy**

| 1 | Epilepsy sub type | 1. Generalized onset 2. Focal onset 3. Unknown Onset |
| --- | --- | --- |
| 2 | Duration since epilepsy diagnosed (in yrs) | ­_________________. |
| 3 | Currently on anti-epileptic treatment | 1. Yes 2. No |
| 4 | Current antiepileptic drugs combination | 1. 0 (Currently not on AEDs 2. 1 (On mono therapy)  3. >2 (On Poly therapy) |
| 5 | Name of a drug/s | __________________________________ |
| 5 | Duration since antiepileptic drugs started (in years) | __________________ . |
| 6 | Drug responsiveness status | 1. Drug responsive 2. Drug-resistant 3. Undefined |

| **Step 3 Physical Measurements** |
| --- |

| **Blood Pressure** | | | |
| --- | --- | --- | --- |
| **Question** | **Response** | | **Code** |
| Interviewer ID |  | └─┴─┘ | M1 |
| Device ID for blood pressure |  | └─┴─┘ | M2 |
| Cuff size used | Small | 1 | M3 |
|  | Medium | 2 |  |
|  | Large | 3 |  |
| Reading 1 | Systolic ( mmHg) | └─┴─┴─┘ | M4a |
|  | Diastolic (mmHg) | └─┴─┴─┘ | M4b |
| Reading 2 | Systolic ( mmHg) | └─┴─┴─┘ | M5a |
|  | Diastolic (mmHg) | └─┴─┴─┘ | M5b |
| Reading 3 | Systolic ( mmHg) | └─┴─┴─┘ | M6a |
|  | Diastolic (mmHg) | └─┴─┴─┘ | M6b |
| During the past two weeks, have you been treated for raised blood pressure with drugs (medication) prescribed by a doctor or other health worker? | Yes | 1 | M7 |
|  | No | 2 |  |
| **Height and Weight** | | | |
| **For women:** Are you pregnant? | Yes | 1 *If Yes, go to M 16 a-c* | M8 |
|  | No | 2 |  |
| Interviewer ID |  | └─┴─┘ | M9 |
| Device IDs for height and weight | Height | └─┴─┘ | M10a |
|  | Weight | └─┴─┘ | M10b |
| Height | in Centimetres (cm) | └─┴─┴─┘. └─┘ | M11 |
| Weight  *If too large for scale 666.6* | in Kilograms (kg) | └─┴─┴─┘.└─┘ | M12 |
| **Waist** | | | |
| Device ID for waist |  | └─┴─┘ | M13 |
| Waist circumference | in Centimetres (cm) | └─┴─┴─┘.└─┘ | M14 |

| **Step 4 Biochemical Measurements** |
| --- |

| **CORE: Blood Glucose** | | | |
| --- | --- | --- | --- |
| **Question** | **Response** | | **Code** |
| During the past 12 hours have you had anything to eat or drink, other than water? | Yes | 1 | B1 |
|  | No | 2 |  |
| Technician ID |  | └─┴─┘ | B2 |
| Device ID |  | └─┴─┘ | B3 |
| Time of day blood specimen taken (24 hour clock) | Hours : minutes | └─┴─┘: └─┴─┘  hrs mins | B4 |
| Fasting blood glucose | mg/dl | └─┴─┴─┘.└─┘ | B5 |
| Today, have you taken insulin or other drugs (medication) that have been prescribed by a doctor or other health worker for raised blood glucose? | Yes | 1 | B6 |
|  | No | 2 |  |
| **CORE: Blood Lipids** | | | |
| Total cholesterol | mg/dl | └─┴─┴─┘.└─┘ | B8 |
| During the past two weeks, have you been treated for raised cholesterol with drugs (medication) prescribed by a doctor or other health worker? | Yes | 1 | B9 |
|  | No | 2 |  |

| **EXPANDED: Triglycerides and HDL Cholesterol** | | | |
| --- | --- | --- | --- |
| **Question** | **Response** | | **Code** |
| HDL Cholesterol | mg/dl | └─┴─┴─┘.└─┘ | B17 |
| TG | (mg/dl) └─┴─┴─┘.└─┘ |  |  |
| LDL-C | mg/dl └─┴─┴─┘.└─┘ |  |  |

**Amharic Version Questioner**

***የኢትዮጲያ STEPS ተላላፊ ላልሆኑ በሽታዎች አጋላጭ ባህሪያትና የተወሰኑ ተላላፊ ላልሆኑ በሽታዎች ስርጭት ዳሰሳ ጥናት የተዘጋጀ መጠይቅ***

*በመጠይቅ ውስጥ ስላሉ ማብራሪያ*

*የሚከተለው ሰንጠረዥ ስለእያንዳንዱ column ስር ስላሉ ጉዳዩች ማብራሪያ የሚሰጥ ይሆናል፡፡*

| **ጥያቄዎች** | እያንዳንዱ ጥያቄ ለጥናቱ ተሳታፊዎች መነበብ አለበት |
| --- | --- |
| **መልስ** | ይህ ክፍል የጥናቱ ተሳታፊዎች የሚሰጡትን መልስ የምንጽፍበት ቦታ ይሆናል፡፡ ጠያቂው መልሶቹ ላይ ማክበብ ወይም ወይም በተሰጠው ባዶ ቦታ ላይ መልሶችን መጻፍ አለበት፡፡ ወደሚቀጥለው እለፍ የሚሉ መመሪያዎችና በመልሶች በኩል የተጻፈ ስለሆነ ጠያቂው ይሄን ሲያይ ወደተገቢው ቦታ ማለፍ ይጠበቅበታል፡፡ |
| **መለያ** | ይህ የእያንዳንዱ ጥያቄ መጠይቅ ቁጥር ሲሆን ቃለመጠይቁን የሚያደርገው ሰው በቀላሉ እንዲያገኛቸው ይረዳዋል |

| **ስለጥናቱ መረጃ** |
| --- |

| *ክፍል 1 የዲሞግራፊክ ጥያቄዎች (ቃለመጠይቅ የሚደረግለት ግለሰቦች ዝርዝር መረጃ)* |
| --- |

| *የዲሞግራፊክ ጥያቄዎች* | | | | |
| --- | --- | --- | --- | --- |
| ጥያቄዎች | መልስ | | | መለያ |
| ጾታ በማየት ይመዝገብ ? | Male | | 1 | C1 |
|  | Female | | 2 |  |
| እድሜህ/ሽ ምን ያህል ነው  በሙሉ አመት ይጻፍ? | Years | | └─┴─┘ | C2 |
| አጠቃላይ በትምህርት ቤት ከጀመርህ/ሽ ጀምሮ ምን ያህል አመት አሳለፍክ/ሽ? | Years | └─┴─┘ | | C3 |

| ተጨማሪ የዲሞግራፊክ ጥያቄዎች | | | |
| --- | --- | --- | --- |
| የትምህርት ደረጃ  እስከ ስንት /ምን/ ድረስ ተምረዋል | No formal schooling | 1 | C4 |
|  |  |  |  |
|  | Primary school completed | 2 |  |
|  | Secondary school completed | 3 |  |
|  | College/University completed | 4 |  |
|  | Post graduate degree | 5 |  |
|  | Refused | 88 |  |
| ከሚከተሉት የትኛዉ የባለፉት 12 ወራተ ዋና የሆነዉን የሥራዎን ሁኔታ ይገልጻል? | Government employee | 1 | C5 |
|  | Non-government Employee | 2 |  |
|  | Private employee | 3 |  |
|  | Farmer | 4 |  |
|  | Trader | 5 |  |
|  | Retired | 6 |  |
|  | Daily labor | 7 |  |
|  | Others | 8 __________________________ |  |
|  | Refused | 88 |  |

| Question | Response | | | Code |
| --- | --- | --- | --- | --- |
| ያለፈውን አንድ አመት ከግምት ውስጥ በማስገባት በአማካኝ የቤትዎ ገቢ ምን ያህል ነበር (አንድን ብቻ ምረጥ) | Per week | └─┴─┴─┴─┴─┴─┴─┘ Go to T1 | | C6a |
|  | OR per month | └─┴─┴─┴─┴─┴─┴─┘ Go to T1 | | C6b |
|  | OR per year | └─┴─┴─┴─┴─┴─┴─┘ Go to T1 | | C6c |
|  | Refused | 88 | | C6d |
| ከላይ የተጠቀሰው ገቢዎ ምን ያህል እንደሆነ ካላወቁ ከሚከተሉት ውስጥ በግምት አመታዊ የቤትዎ ገቢ ምን ያህል ነው?  (ምርጫውን አንብብላቸው የቤተሰብዎ ጠቅላላ ገቢ) | ≤ 12,000 Birr | | 1 | C7 |
|  | More than 12,000 ≤ 18,000 Birr | | 2 |  |
|  | More than 18,000 ≤23,300 | | 3 |  |
|  | More than 23,300 ≤ 30,000 | | 4 |  |
|  | More than 30,000 | | 5 |  |
|  | Don't Know | | 77 |  |
|  | Refused | | 88 |  |

| ***ክፍል 1 ባህሪን የሚለኩ መጠይቆች*** |
| --- |

| ሲጋራን ስለመጠቀም /ሲጋራ ማጨስን በተመለከተ/ | | | |
| --- | --- | --- | --- |
| ሲጋራን ስለመጠቀም /ሲጋራ ማጨስን በተመለከተ/  አሁን በማስከተል ከጤና ጋር ተያያዥነት ስላላቸው የተለያዩ ባሕሪያት እጠይቅዎታለሁ፡፡ ከዚህጀም መካከል ስለማጨስ አልኮል ስለመጠጣት አትክልትና ፍራፍሬን መመገብን እና የአካል ብቃት እንቅስቃሴን ይመለከታል፡፡ ለመጀመር ያህል እስኪ ሲጋራን ማጨስን በተመለከተ የተወሰኑ ጥያቄዎችን ላቅርብልዎ፡፡ | | | |
| ጥያቄዎች | መልስ | | መለያ |
| በአሁኑ ሰአት ሲጋራ፣ጋያ የመሳሰሉትን ያጨሳሉ? (ካርዱን ያሳዩአቸው) | Yes | 1 | T1 |
|  | No | 2 *If No, go to T8* |  |
| በአሁኑ ሰአት ሲጋራ በየቀኑ ያጨሳሉ? | Yes | 1 | T2 |
|  | No | 2 |  |
| ማጨስ የጀመሩት በስንት አመትዎ ነው? | Age (years) | **└─┴─┘** *If Known, go to T5a/T5aw* | T3 |
|  | Don’t know 77 |  |  |
| ማጨስ ከጀመሩ ስንት አመት በፊት እንደሆነ ያስታውሳሉ?  (አንድ ብቻ ሙላ) | In Years | **└─┴─┘** *If Known, go to T5a/T5aw* | T4a |
|  | OR in Months | **└─┴─┘** *If Known, go to T5a/T5aw* | T4b |
|  | OR in Weeks | **└─┴─┘** | T4c |
| ከሚከተሉት የሲጋራ አይነቶች የትኛውን ነው በየቀኑ የሚጠቀሙት? (ካርዱን ያሳዩአቸው እያንዳንዱን አይነት ጥቀስ)  አላዉቅም 7777 | DAILY**↓** WEEKLY↓ | | |
|  | Manufactured cigarettes | └─┴─┴─┴**─┘**└─┴─┴─┴**─┘** | T5a/T5aw |
|  | Hand-rolled cigarettes | └─┴─┴─┴**─┘**└─┴─┴─┴**─┘** | T5b/T5bw |
|  | Pipes full of tobacco | └─┴─┴─┴**─┘**└─┴─┴─┴**─┘** | T5c/T5cw |
|  | Number of Shisha sessions | └─┴─┴─┴**─┘**└─┴─┴─┴**─┘** | T5e/T5ew |
|  | Gaya | └─┴─┴─┴**─┘**└─┴─┴─┴**─┘** | T5X/  T5XWw |
|  | Other | └─┴─┴─┴**─┘**└─┴─┴─┴**─┘** *If Other, go to T5other, else go to T6* | T5f/T5fw |
|  | Other (please specify): | _______________________________ | T5other/  T5otherw |
| ባለፋት 12 ወራት ሲጋራ ማጨስ ለማቆም ጥረት አድረገዉ ነበር? | Yes | 1 | T6 |
|  | No | 2 |  |
| ባለፋት 12 ወራት በማንኛዉም ጉብኝት ወቅት ት ሲጋራ ማጨስ እንዲያቆሙ በጤና ባለሙያ ተመክረዉ ነበር? | Yes | 1 *If T2=Yes, go to T12; if T2=No, go* *to T9* | T7 |
|  | No | 2 *If T2=Yes, go to T12; if T2=No, go to T9* |  |
|  | No visit during the past 12 months | 3 *If T2=Yes, go to T12; if T2=No, go to T9* |  |
| ከዚህ በፍት ሲጋራ አጭሰዉ ያዉቃሉ?  (ካርዱን ያሳዩአቸው) | Yes | 1 | T8 |
|  | No | 2 *If No, go to T12* |  |
| ከዚህ በፍት በየቀኑ ሲጋራአጭሰዉ ያዉቃሉ? | Yes | 1 *If T1=Yes, go to T12, else go to T10* | T9 |
|  | No | 2 *If T1=Yes, go to T12, else go to T10* |  |

| ማጨስ ሲያቆሙ እድሜዎ ስንት ነበር? | Age (years) | **└─┴─┘** *If Known, go to T12* | T10 |
| --- | --- | --- | --- |
|  | Don’t Know 77 |  |  |
| ማጨስ ካቆሙ ምን ያህል ጊዜ ሆንዎት??  (አንዱን ብቻ ሙላ1)  አላዉቅም 77 | Years ago | **└─┴─┘** *If Known, go to T12* | T11a |
|  | OR Months ago | **└─┴─┘** *If Known, go to T12* | T11b |
|  | OR Weeks ago | **└─┴─┘** | T11c |

| **ሲጋራን ስለመጠቀም /ሲጋራ ማጨስን በተመለከተ/** | | | |
| --- | --- | --- | --- |
| ጥያቄዎች | መልስ | | መለያ |
| በአሁኑ ሰአት ጭስ አልባ የሆነ ሲጋራ ይጠቀማሉ? /ለምሳሌ የሚታኘከው ሲጋራ፤ ሱረት(ካርዱን ያሳዩአቸው) | Yes | 1 | T12 |
|  | No | 2 *If No, go to T15* |  |
| በአሁኑ ሰአት ጭስ አልባ የሆኑ ሲጋራዎችን በየእለቱ ይጠቀማሉ? | Yes | 1 | T13 |
|  | No | 2 *If No, go to T14aw* |  |
| በአማካኝ በየቀኑ ምን ያህል ጭስ አልባ የሆኑ ሲጋራዎች ይጠቀማሉ? (እያንዳንዱ አይነት ይመዝገብ ካርዱን ያሳዩቸው)  *አላዉቅም 7777* | DAILY**↓** WEEKLY↓ | | |
|  | Snuff, by mouth | └─┴─┴─┴**─┘**└─┴─┴─┴**─┘** | T14a/  T14aw |
|  | Snuff, by nose | └─┴─┴─┴**─┘**└─┴─┴─┴**─┘** | T14b/  T14bw |
|  | Chewing tobacco | └─┴─┴─┴**─┘**└─┴─┴─┴**─┘** | T14c/  T14cw |
|  | Other | └─┴─┴─┴**─┘**└─┴─┴─┴**─┘** *If Other, go to T14other, if T13=No, go to T16, else go to T17* | T14e/  T14ew |
|  | Other (please specify): | *If T13=No, go to T16, else go to T17* | T14other/  T14otherw |
| ከዚህ በፍት ጭስ አልባ የሆነ ሲጋራ ይጠቀማሉ? /ለምሳሌ የሚታኘከው ሲጋራ፤ ሱረት(ካርዱን ያሳዩአቸው) | Yes | 1 | T15 |
|  | No | 2 *If No, go to T17* |  |
| ከዚህ በፍት ጭስ አልባ የሆነ ሲጋራ በየቀኑ ይጠቀማሉ? /ለምሳሌ የሚታኘከው ሲጋራ፤ ሱረት(ካርዱን ያሳዩአቸው) | Yes | 1 | T16 |
|  | No | 2 |  |
| ባለፋት 30 ቀናት በቤትዎ ውስጥ ሲጋራ የሚያጨስ ሰዉ ነበር? | Yes | 1 | T17 |
|  | No | 2 |  |
| ባለፋት 30 ቀናት ዉስጥ የአየር እጠርት ባለባቸዉ ዝግ በሆኑ ስፍራዎች ፤የስራ ቦታ (ቢሮ) ሲጋራ የሚያጨስ ሰዉ ነበር? | Yes | 1 | T18 |
|  | No | 2 |  |
|  | Don't work in a closed area | 3 |  |

| **አልኮል መጠጣትን በተመለከተ** | | | |
| --- | --- | --- | --- |
| አሁን ደግሞ ስለአልኮል መጠጣትን በተመለከተ እጠይቀዎታለሁ፡፡ | | | |
| ጥያቄዎች | መልስ | | መለያ |
| አልኮልነት ያላቸው መጠጦች ጠጥተው ያውቃሉ /ለምሳሌ ሲራ ወይን ጠላ የመሳሰሉት ካርዱን ካርዱን አሳያቸው/ | Yes | 1 | A1 |
|  | No | 2  *If No, go to A16* |  |
| ባለፋት 12 ወራት ውስጥ አልኮልነት ያላቸውን መጠጦች ጠጥተዋል? | Yes | 1  *If Yes, go to A4* | A2 |
|  | No | 2 |  |
| በጤናዎ መታወክ ምክንያት ወይም በሀክም ምክር አልኮልነት ያላቸውን መጠጦች መጠጣት አቁመዉ ነበር ? | Yes | 1 *If Yes, go to A16* | A3 |
|  | No | 2 *If No, go to A16* |  |
| ባለፋት 12 ወራት ውስጥ አልኮልነት ያላቸውን በየስንት ጊዘ አንድ ስታነዳርድ መጠጦች ጠጥተው ነበር??  (መልሱን ያንብቡላቸው)  *(USE SHOWCARD)* | Daily | 1 | A4 |
|  | 5-6 days per week | 2 |  |
|  | 3-4 days per week | 3 |  |
|  | 1-2 days per week | 4 |  |
|  | 1-3 days per month | 5 |  |
|  | Less than once a month | 6 |  |
| ባለፋት 30 ቀናት ውስጥ አልኮልነት ያላቸውን መጠጦች ጠጥተው ነበር? | Yes | 1 | A5 |
|  | No | 2 *If No, go to A13* |  |
| ባለፋት 30 ቀናት በየስንት ጊዜው አንድ ስታነዳርድ ይጠጡ ነበር?? | Number  Don't know 77 | └─┴─┘ | A6 |
| ባለፋት 30 ቀናት ውስጥ አልኮል በሚጠጡበት ሰአት በአማካይ በአንድ ጊዜ ስንት ስታነዳርድ አልኮል ይጠጡ ነበር?  (USE SHOWCARD) | Number  Don't know 77 | └─┴─┘ | A7 |
| ባለፋት 30 ቀናት ውስጥ በአንዴ ትልቁ የጠጡት ስንት ስታነዳርድ አልኮል ነበር ሁሉንም ቁጠር | Largest number  Don't Know 77 | └─┴─┘ | A8 |
| ባለፋት 30 ቀናት ውስጥ በአንዴ ከ5-6 ስታነዳርድ አልኮል ስንት ግዘ ወስደዉ ነበርነበር? | Number of times Don't Know 77 | └─┴─┘ | A9 |
| ባለፋት 30 ቀናት ውስጥ አልኮል ሲወስዱ ምን ያህሉን ጊዜ ከምግብ ጋር ይወስዳሉ? | Usually with meals 1  Sometimes with meals 2  Rarely with meals 3  Never with meals 4 | | X6 |
| *ባለፋት 7 ቀናት ውስጥ ስንት አይነት የአልኮል አይነቶችን በየቀኑ ወስደዋል?*  *(USE SHOWCARD)*  *አላዉቅም 77* | Monday | └─┴─┘ | A10a |
|  | Tuesday | └─┴─┘ | A10b |
|  | Wednesday | └─┴─┘ | A10c |
|  | Thursday | └─┴─┘ | A10d |
|  | Friday | └─┴─┘ | A10e |
|  | Saturday | └─┴─┘ | A10f |
|  | Sunday | └─┴─┘ | A10g |

| **አልኮል መጠጣትን በተመለከተ** | | | |
| --- | --- | --- | --- |
| እስካሁን የባለፉት 7 ቀናት የአልኮል አጠቃቀም የተመለከቱ መጠይቆችን ተመልክተናል፡፡ እነዚህ መጠይቆች ጠቅለል ያሉ ናቸዉ ነገር ግን ቀጥሎ በቤት ዉስጥ ስለተዘጋጁ አልኮሎች፤ ከዉጭ አገር ስለመጡ አልኮሎች ወይም ማንኛዉንም አልኮል ለመጠጥ አገልግሎት የማይዉል ወይም ከታክስ ዉጭ ስለገቡ አልኮሎች እጠይቀዎታለሁ፡፡ | | | |
| ጥያቄዎች | መልስ | | መለያ |
| ባለፋት 7 ቀናት ውስጥ አልኮልነት ያላቸውን መጠጦች ጠጥተዋል??  *(USE SHOWCARD)* | Yes | 1 | A11 |
|  | No | 2 *If No, go to A13* |  |
| ባለፋት 30 ቀናት ውስጥ አልኮል በሚጠጡበት ሰአት በአማካይ በአንድ ጊዜ ስንት ስታነዳርድ አልኮል ይጠጡ ነበር??  *(USE SHOWCARD)*  *አላዉቅም 77* | Homebrewed spirits, e.g. Katikala | **└─┴─┘** | A12a |
|  | Homebrewed beer or wine, e.g. Tella, Tej | **└─┴─┘** | A12b |
|  | Alcohol brought over the border/from another country | **└─┴─┘** | A12c |
|  | Alcohol not intended for drinking, e.g. alcohol-based medicines, perfumes, after shaves | **└─┴─┘** | A12d |
|  | Other untaxed alcohol in the country | **└─┴─┘** | A12e |
| ባለፋት 12 ወራት ጊዜ ውስጥ መጠጥ ለማቆም የተቸገሩበት ጊዜ ስንት ነበር?  /መልሱን ያንብቡላቸው/ | Daily or almost daily | 1 | A13 |
|  | Weekly | 2 |  |
|  | Monthly | 3 |  |
|  | Less than monthly | 4 |  |
|  | Never | 5 |  |
| ባለፋት 12 ወራት ጊዜ ውስጥ ከመጠጥ የተነሳ የሚጠበቅቦትዎን ያልተወጡበት ጊዜ ስንት ነበር? | Daily or almost daily | 1 | A14 |
|  | Weekly | 2 |  |
|  | Monthly | 3 |  |
|  | Less than monthly | 4 |  |
|  | Never | 5 |  |
| ባለፋት 12 ወራት ጊዜ ውስጥ በጠዋት የጠጡበት ስንት ጊዜ ነበር? | Daily or almost daily | 1 | A15 |
|  | Weekly | 2 |  |
|  | Monthly | 3 |  |
|  | Less than monthly | 4 |  |
|  | Never | 5 |  |
| ባለፋት 12 ወራት ጊዜ ውስጥ በበተሰብዎ ዉስጥ እርስዎ ወይም ሌላ ሰዉ አልኮል ከመጠጣቱ የተነሳ ችግር ተፈጢሮ ያዉቅ ነበር? | Yes, more than monthly | 1 | A16 |
|  | Yes, monthly | 2 |  |
|  | Yes, several times but less than monthly | 3 |  |
|  | Yes, once or twice | 4 |  |
|  | No | 5 |  |

| ***ጫት ስለመቃም.)*** | | | | | |
| --- | --- | --- | --- | --- | --- |
| ጫት ስለመቃም. | | | | | |
| **ጥያቄ** | **መልስ** | | | **መለያ** | |
| ጫት ቅመው ያውቃሉ?  *(USE SHOWCARD)* | | Yes | 1 | | K1 |
|  |  | No | 2 *If No, go to K14* | |  |
| አሁን ጫት ይቅማሉ? | | Yes | 1 | | K2 |
|  |  | No | 2 If No, go to K8 | |  |
| ባለፋት 12 ወራት ውስጥ በየስንት ግዜ ጫት ይቅማሉ? | | Daily  5-6 days per week  3-4 days per week  1-2 days per week  1-3 days per month  Less than once a month | 1  2  3  4  5  6 | | K3 |
| መቃም የጀመሩት በስንት አመትዎ ነው? | | Age (years) **└─┴─┘** | *If Known, go to K5* | | K4 |
|  |  | Don’t know 77 |  |  |  |
| መቃም የጀመሩት ከምን ያህል ግዜ በፍት እንደሆነ ያስታዉሳሉ ?  *(እንዱን ብቻ አክብብ፣)*  *አላዉቅም 77* | | In Years | **└─┴─┘** *If Known, go to K5* | | K4a |
|  |  | OR in Months | **└─┴─┘** *If Known, go to K5* | | K4b |
|  |  | OR in Weeks | **└─┴─┘** | | K4c |
| *በአማካይ ስንት ዞርባ ጫት በቀን/በሳምንት ይቅማሉ?*  *(ከቀናት ትንሽ ከሆኔ በሳምንት ይሞላ)*  *(USE SHOWCARD)*  *አላዉቅም 77* | | DAILY**↓** WEEKLY↓ | | | |
|  |  | Bundles of Khat | └─┴**─┘** └─┴**─┘** | | K5 |
| ባለፋት 12 ወራት ጫት መቃም ለማቆም ጥረት አድረገዉ ነበር? | | Yes | 1 | | K6 |
|  |  | No | 2 | |  |
| ባለፋት 12 ወራት በጤና ተቃም ወይም ባለሙያ ጉብኝ ወቅት ጫት መቃም እንድያቆሙ ተመክረዉ ያዉቃሉ? | | Yes | 1 go to K9 | | K7 |
|  |  | No | 2 *go* *to K9* | |  |
|  |  | No visit during the past 12 months | 3 *go* *to K9* | |  |
| ጫት መቃም ያቆሙት በስንት አመትዎ ነው? | Age (years) | | **└─┴─┘** *If Known, go to K10* | K8 | |
| ጫት መቃም ካቆሙ ስንት ግዜ ሆኗል?  (አንዱን ብቻ አክብብ 1)  አላዉቅም 77 | Years ago | | **└─┴─┘ If Known, go to K10** | K8a | |
|  | OR Months ago | | **└─┴─┘ If Known, go to K10** | K8b | |
|  | OR Weeks ago | | **└─┴─┘if known, go to K10** | K8c | |
| አሁን ሲቅሙ ሲጋራ ያጨሳሉ??  (USE TOBACCO SHOWCARD) | Yes  No | | 1 If yes, go to K11  2 | K9 | |
| ከዚህ በፍት ሲቅሙ ሲጋራ ያጨሳሉ? | Yes  No | | 1 If K2=2 go to K13  2 If K2=2 go to K13 | K10 | |
| ጫት በሚቅሙ ጓደኛዎ አንዱ ሲጋራ ያጨሳል? | Yes  No | | 1  2 | K11 | |
| ጫት ከቃሙ በኋላ መጠጥ ጠጥተዉ ያዉቃሉ? | Yes  No | | 1 If yes, go to K14 | K12 | |
| ከዚህ ጫት ከቃሙ በኋላ መጠጥ ጠጥተዉ ያዉቃሉ? | Yes  No | | 1  2 | K13 | |
| ባለፋት 12 ወራት ጊዜ ውስጥ በበተሰብዎ ዉስጥ እርስዎ ወይም ሌላ ሰዉ ጫት ከመቃሙ የተነሳ ችግር ተፈጢሮ ያዉቅ ነበር? | Yes  No | | 1  2 | K14 | |

| **ስነ ምግብ** | | | |
| --- | --- | --- | --- |
| ቀጥሎ የምንጠይቅዎ በብዛት ስለሚወስዱት አትክልት እና ፍራፍሬ ይሆናል፡፡ በአካባቢዎ በብዛት ስላሉት አትክልት እና ፍራፍሬዎች የሚያሳይ ካርድ አሳይዎታለሁ፡፡ እያንዳንዱ ስእል የሚወክለው የሚወሰዱትን የምግብ መጠን ይሆናል መልስ በሚሰጡኝ ሰአት ባለፈው አመት ምግቡን የወሰዱበትን ሳምንት እያስታወሱ እንዲሆን እጠይቅዎታለሁ፡፡ | | | |
| **ጥያቄዎች** | **መልስ** | | **መለያ** |
| በሳምንት ምን ያህል ቀን ፍራፍሬ ይወስዳሉ? (ካርዱን ያሳዩአቸው) | Number of days Don't Know 77 | └─┴─┘ If Zero days, go to D3 | D1 |
| ከተጠቀሱት ቀናት ዉስጥ በኣንድ ቀን ምን ያህል የፍራፍሬ መጠን ወስደዋል? (ካርዱን ያሳዩአቸው) | Number of servings  Don't Know 77 | └─┴─┘ | D2 |
| በሳምንት ምን ያህል ቀን አትክልት ይመገባሉ? /ካርዱን ያሳዩአቸው/ | Number of days Don't Know 77 | └─┴─┘ If Zero days, go to D5 | D3 |
| አትክልት በወሰዱባቸው ቀናት ምን ያህል ጊዜ በቀን ውስጥ አትክልት ወስደዋል? (ካርዱን ያሳዩአቸው) | Number of servings  Don’t know 77 | └─┴─┘ | D4 |

| ***የአካል ብቃት እንቅስቃሴ በተመለከተ*** | | | |
| --- | --- | --- | --- |
| ቀጥሎ በሳምንት ምን ያህል ጊዜ ለአካል ብቃት እንቅስቃሴዎች እንደሚያዘወትሩ እጠይቅዎታለሁ፡፡ ምንም እንኳ ጠንከር ያለ የአካል ብቃት እንቅስቃሴ አረጋለሁ ብለው ቢያስቡም ጥያቄዎቹን ለመመለስ ጥረት ያድርጉ፡፡ በመጀመሪያ ለሥራ ምን ያህል ጊዜ እንደሚያጠፋ ያስቡ፡፡ ተከፍሉት ወይም ሳይከፈልዎት ስለሚሰሩት ስራም እያሰቡ ይነግሩኛል፡፡ እነዚህም ሥራዎች እንደ ትምህርት እርሻ አሳ ማጥመድ አደን የመሳሰሉትን ሊሆኑ ይችላሉ፡፡ የሚከተሉትን ጥይቄዎች ሲመልሱልኝ ከበድ ያለ እንቅስቃሴ ስንል ከፍተኛ ጉልበት የሚፈልጉና በልብ ምትና አተነፋፈስ ሁኔታና በከፍተኛ ደረጃ የሚጨምሩ ናቸው፡፡ መካከለኛ እንቅስቃሴ ስንል መካከለኛ መጠነኛ የሆነ ጉልበት የሚፈልጉና በተወሰነ መጠን ብቻ የልብ እና አተነፋፈስ መጠንን የሚጨምሩ ናቸው፡፡ | | | |
| **ጥያቄዎች** | **መልስ** | | **መለያ** |
| **ሥራ** | | | |
| የሚሰሩት ስራ ከባድ እንቅስቃሴ የሚያስፈልገውና የልብ ምትዎና አተነፋፈስዎና በከፍተኛ ሁኔታ የሚጨምሩ ናቸው /እንደምሳሌ ከባድ ነገሮችን መሸከም መጫን መቆፈር ከ10 ደቂቃ በላይ በተከታታይ ግንባታ ሊሆኑ ይችላሉ/  *(USE SHOWCARD)* | Yes | 1 | P1 |
|  | No | 2  *If No, go to P 4* |  |
| በሳምንት ለምን ያህል ቀናት እነዚህን ከበድ ያሉ ሥራዎች ይሰራሉ?? | Number of days | └─┘ | P2 |
| በቀን ውስጥ ምን ያህል ሰአታት እነዚህን ከበድ ያሉ ሥራዎች ይሰራሉ? | Hours : minutes | └─┴─┘: └─┴─┘  hrs mins | P3 (a-b) |
| የምትሰራው ሥራ መጠነኛ የሆነ እንቅስቃሴ የሚያስፈልገውና በመጠኑ የልብ ምት እና አተነፋፈስን የሚጨምሩ ናቸው፡፡ ለምሳሌ እንደ እርምጃ ሊሆን ይችላል?  *(USE SHOWCARD)* | Yes | 1 | P4 |
|  | No | 2 *If No, go to P 7* |  |
| በሳምንት ለምን ያህል ቀናት መጠነኛ እንቅስቃሴ የሚወስዱ ስራዎች *ይሰራሉ*? | Number of days | └─┘ | P5 |
| በቀን ውስጥ ምን ያህል ሰአታት መጠነና እንቅስቃሴ የሚወስዱ ስራዎች ይሰራሉ? | Hours : minutes | └─┴─┘: └─┴─┘  hrs mins | P6 (a-b) |
| **ከቦታ ቦታ የሚንቀሳቀሱባቸውን ዘዴዎች በተ8መለከተ (መጓጓዣን)** | | | |
| ቀጥለው ያሉ ጥያቄዎች በመደበኛነት በስራ ቦታዎ ላይ ስለሚያደርጉት እንቅስቃሴና ከላይ የተጠቀሱትን አያካትትም፡፡ አሁን የምንጠይቅዎ በመደበኛነት ከቦታ ቦታ ስለሚጓጓዙበት ይሆናል /ለዚህም እንደምሳሌ ወደ ስራ ቦታ ወደ ሱቅ ወደመሳሰሉት ቦታ የሚሄዱበትን መንገድ ይሆናል፡፡ | | | |
| ከቦታ ቦታ ለመሄድ በእግርዎ ወይም በሳይክል ቢያንስ ለ10 ደቂቃ በየቀኑ ይጓዛሉ? | Yes | 1 | P7 |
|  | No | 2  *If No, go to P 10* |  |
| በሳምንቱ ውስጥ ምን ያህል ቀን በእግር ወይም በሳይክል ቢያንስ ለ10 ደቂቃ ያህል ከቦታ ወደ ቦታ ይሄዳሉ? | Number of days | └─┘ | P8 |
| በቀን ውስጥ ለምን ያህል ሰአት በእግርዎ ወይም በሳይክል ይጓዛሉ | Hours : minutes | └─┴─┘: └─┴─┘  hrs mins | P9 (a-b) |

| **ከመዝናናት እንቅስቃሴዎች ጋር በተያያዘ ያለ ጥያቄ** | | | |
| --- | --- | --- | --- |
| የሚከተሉት ጥያቄዎች ከላይ ለስራ ወይም ለመጓጓዝ የጠቀሷቸውን አያካትቱም አሁን መጠየቅ የምፈልገው ከስፖርት ጂምናዚየም ዋና ስለመሳሰሉት የሚያደርጉትን እንቅስቃሴዎች በተመለከተ ይሆናል፡፡. | | | |
| ከበድ ያለ እንቅስቃሴን የሚፈልጉ ስፖርቶች ጂምናዚየም /ለምሳሌ ሩጫ ወይም ኳስ ጨዋታ ሊሆን ይችላል ቢያንስ ለ10 ደቂቃ በተከታታይ ይሰራሉ? *(USE SHOWCARD)* | Yes | 1 | P10 |
|  | No | 2  *If No, go to P 13* |  |
| በሳምንት ለምን ያህል ቀን እነዚህን ከበድ ያለ እንቅስቃሴ የሚፈልጉ ስፖርቶች ይሰራሉ? | Number of days | └─┘ | P11 |
| በቀን ውስጥ ለምን ያህል ሰአት እነዚህን ከበድ ያሉ እንቅስቃሴ ስፖርቶች ይሰራሉ? | Hours : minutes | └─┴─┘: └─┴─┘  hrs mins | P12  (a-b) |

| ***አካል ብቃት እንቅስቃሴ በተመለከተ የቀጠለ…*** | | | |
| --- | --- | --- | --- |
| **ጥያቄዎች** | **መልስ** | | **መለያ** |
| ቀለል ያለ እንቅስቃሴ የሚፈሉጉ ስፖርቶች ጂምናዚየሞች /ለምሳሌ እንደ ዋና የእጅ ኳስ ሳይክል መንዳት/ የመሳሰሉትን ቢያንስ ለ10 ደቂያ በተከታታይ ይሰራሉ?  (USE *SHOWCARD)* | Yes | 1 | P13 |
|  | No | 2  *If No, go to P16* |  |
| በሳምንት ለምን ያህል ቀን እነዚህን ቀለል ያለ እንቅስቃሴ የሚፈልጉ ስፖርቶች ይሰራሉ? | Number of days | └─┘ | P14 |
| በቀን ውስጥ ለምን ያህል ሰአት እነዚህን ቀለል ያለ እንቅስቃሴ የሚፈልጉ ስፖርቶች ይሰራሉ? | Hours : minutes | └─┴─┘: └─┴─┘  hrs mins | P15 (a-b) |

| ***አካል ብቃት እንቅስቃሴ*** | | | |
| --- | --- | --- | --- |
| *ስለዝግተኛ የሆነ አካል ብቃት እንቅስቃሴ ባሕሪን በተመለከተ*  **)** | | | |
| የሚከተሉትን ጥያቄዎች በቤት ወይም በስራ በታ በመቀመጥ ስለሚወስደው ሰአት ከጓደኛዎት ጋር በመቀመጥ ስለሚወስድበት ጊዜ በመኪና በፈረስ ሲግዙ የሚፈጅበትን ሰአት እንዲሁም ቴሌቪዥን በመከታታል ካርታ በመጫወት የሚፈጅበትን ሰአት ቢነግረኝ፡፡ በእንቅልፍ የሚያሳልፋትን ሰአት አያካትትም.  *(USE SHOWCARD)* | | | |
| በቀን ውስጥ ምን ያህል ሰአት ከላይ ለጠቀስኳቸው ነገሮች በመቀመጥ ያሳልፋሉ?? | Hours : minutes | └─┴─┘: └─┴─┘  hrs mins | P16  (a-b) |

| **የደም ግፊት መጨመር የሚመለከቱ ጥያቄዎች** | | | |
| --- | --- | --- | --- |
| **ጥያቄ** | **መልስ** | | **መለያ** |
| ቀደም ባለ ጊዜ በሀኪም ወይም በሌላ የጤና ባለሙያ የደም ግፊትዎን ተለክተው ያውቃሉ? | Yes | 1 | H1 |
|  | No | 2 *If No, go to X10* |  |
| ቀደም ባለ ጊዜ በሀኪም ወይም በሌላ የጤና ባለሙያ የደም ግፊቶ ጨምሮአል ወይም የደም ግፊት ህመም እንዳለቦት ተነግሮት ያውቃሉ? | Yes | 1 | H2a |
|  | No | 2  *If No, go to X10* |  |
| ባለፉት 12 ወራት ውስጥ ይህ በሽታ እንዳለቦት ተነግሮት ያውቃሉ? | Yes | 1 | H2b |
|  | No | 2 |  |
| ባለፉት 2 ሳምንታት ውስጥ በሀኪም ወይም በሌላ የጤና ባለሙያ የታዘዘ ማንኛውም ዐይነት የደም ግፊት መድሃኒት ወስደዋል? | Yes | 1 | H3 |
|  | No | 2 |  |
| የደም ግፊትዎን ለመታከም የባህል ሀኪም ጋር ሄደው ያውቃሉ? | Yes | 1 | H4 |
|  | No | 2 |  |
| ባሁኑ ሰዐት የደም ግፊትዎን ለማዳን እየወሰዱት ያለ የባህል ህክምና ወይም የተፈጥሮ መድሃኒት አለ? | Yes | 1 | H5 |
|  | No | 2 |  |
| በቤተሰቦዎ(እናት፥ አባት፥ ወንድም፥ እሀት ወይም ልጆች) ውስጥ የደም ግፊት መጨመር ወይም የደም ግፊት ህመም ያለበት ሰው እለ? | Yes | 1 | X10 |
|  | No | 2 |  |

| **የስኳር በሽታን የሚመለከቱ ጥያቄዎች** | | | |
| --- | --- | --- | --- |
| ቀደም ባለ ጊዜ በሀኪም ወይም በሌላ የጤና ባለሙያ የደም ውስጥ ስኳር መጠን ተለክተው/ተመርምረው ያውቃሉ? | Yes | 1 | H6 |
|  | No | 2 *If No, go to X11* |  |
| ቀደም ባለ ጊዜ በሀኪም ወይም በሌላ የጤና ባለሙያ በደም ውስጥ የስኳር መጠን ጨምሮአል ወይም የስኳር በሽታ እንዳለቦት ተነግሮት ያውቃሉ? | Yes | 1 | H7a |
|  | No | 2  *If No, go to X11* |  |
| ባለፉት 12 ወራት ይህ በሽታ እንዳለቦት ተነግሮት ያውቃሉ? | Yes | 1 | H7b |
|  | No | 2 |  |
| ባለፉት 2 ሳምንታት ውስጥ በሀኪም ወይም በሌላ የጤና ባለሙያ የታዘዘ ማንኛውም ዐይነት የስኳር መድሃኒት ወስደዋል? | Yes | 1 | H8 |
|  | No | 2 |  |
| ባሁኑ ሰዐት በሀኪም ወይም በሌላ የጤና ባለሙያ የታዘዘ የኢንሱሊን መርፌ(Insulin) ለስኳር ህመሞ እየወሰዱ ነው? | Yes | 1 | H9 |
|  | No | 2 |  |
| የስኳር በሽታን ለመታከም የባህል ሀኪም ጋር ሄደው ያውቃሉ? | Yes | 1 | H10 |
|  | No | 2 |  |
| ባሁኑ ሰዐት የስኳር በሽታዎን ለማዳን እየወሰዱት ያለ የባህል ህክምና ወይም የተፈጥሮ መድሃኒት አለ? | Yes | 1 | H11 |
|  | No | 2 |  |
| በቤተሰቦዎ(እናት፥ አባት፥ ወንድም፥ እሀት ወይም ልጆች) ውስጥ በደም ውስጥ የስኳር መጠን መጨመር ወይም የስኳር በሽታ ያለበት ሰው እለ? |  | Yes 1  No 2 | X11 |

| **የኮሌስትሮል(የደም ውስጥ ስብ/ቅባት) መጠን መጨመር** **የሚመለከቱ ጥያቄዎች** | | | | |
| --- | --- | --- | --- | --- |
| **ጥያቄ** | **መልስ** | | | **መለያ** |
| ቀደም ባለ ጊዜ በሀኪም ወይም በሌላ የጤና ባለሙያ ኮሌስትሮል(የደም ውስጥ ስብ/ቅባት) መጠን ተለክተው/ተመርምረው ያውቃሉ? | Yes | 1 | H12 | |
|  | No | 2 *If No, go to X12* |  |  |
| ቀደም ባለ ጊዜ በሀኪም ወይም በሌላ የጤና ባለሙያ የኮሌስትሮል(የደም ውስጥ ስብ/ቅባት) መጠን ጨምሮእል ተብሎ ተነግሮት ያውቃሉ? | Yes | 1 | H13a | |
|  | No | 2 *If No, go to X12* |  |  |
| ባለፉት 12 ወራት ይህ በሽታ እንዳለቦት ተነግሮት ያውቃሉ? | Yes | 1 | H13b | |
|  | No | 2 |  |  |
| ባለፉት 2 ሳምንታት ውስጥ በሀኪም ወይም በሌላ የጤና ባለሙያ የታዘዘ ማንኛውም ዐይነት የኮሌስትሮል መጨመር መድሃኒት ወስደዋል? | Yes | 1 | H14 | |
|  | No | 2 |  |  |
| የኮሌስትሮል መጨመርን ለመታከም የባህል ሀኪም ጋር ሄደው ያውቃሉ? | Yes | 1 | H15 | |
|  | No | 2 | |  |
| ባሁኑ ሰዐት የኮሌስትሮል መጨመርን ለማዳን እየወሰዱት ያለ የባህል ህክምና ወይም የተፈጥሮ መድሃኒት አለ? | Yes | 1 | | H16 |
|  | No | 2 | |  |
| በቤተሰቦዎ(እናት፥ አባት፥ ወንድም፥ እሀት ወይም ልጆች) ውስጥ የኮሌስትሮል መጨመር ያለበት ሰው እለ? | Y  N | 1  2 | | X12 |

| **ከልብ በሽታ ጋር የሚገናኙ ጥያቄዎች** | | | |
| --- | --- | --- | --- |
| ቀደም ባለ ጊዜ በሀኪም ወይም በሌላ የጤና ባለሙያ የልብ ድካም፥ ከልብ ጋር የተገናኘ በደረት ላይ ከባድ የዉጋት እና የአየር ማጣት ስሜት (angina) ወይም ጭንቅላት ውስጥ ደም መፍሰስ/መርጋት እንደደረሰቦት  ተነግሮት ያውቃሉ? | Yes | 1 | H17 |
|  | No | 2 |  |
| ባሁኑ ሰዐት በየቀኑ አስፕሪን(Aspirin) ለልብ በሽታ እንደህክምና ወይም እንደመከላከያ እየወሰዱት ነው? | Yes | 1 | H18 |
|  | No | 2 |  |
| ባሁኑ ሰዐት በየቀኑ አስታቲን[ሎቫአስታቲን(Lovastatin)፣ ሲምቫአስታቲን(Simvastatin)፣ አትሮቫአስታቲን(Atorvastatin)ወይም ሌላ የአስታቲን ዐይነት] ለልብ በሽታ እንደህክምና ወይም እንደመከላከያ እየወሰዱት ነው? | Yes | 1 | H19 |
|  | No | 2 |  |

**የሚጥል በሽታን ክሊኒካዊ ባህሪያትን እና ሕክምናን የሚመለከቱ መጠይቆች**

| 1 | የሚጥል በሽታ ዓይነት | 1. Generalized onset 2. Focal onset 3. Unclassified |
| --- | --- | --- |
| 2 | በሽታዉ ከጀመረዎት ምን ያህል ጊዜ ሆነዎት? | በአመት____________, በወር__________________________ |
| 3 | አሁን ላይ እየዎሰዱት ያለ የሚጥል በሽታ መድኃኒት/ቶች አጠቃቀም / ጥምረት | 1. ያልጀመረ 2. አንድ መዲሃኒት  3. ሁለት መዲሃኒት 4. ከሁለት በላይ |
| 4 | የመድሃኒቱ ስም | 1. Carbamazepine 2. Phenytoin 3. Valproate  4. Phenobarbitone 5. ሌላ (ይግለጹ)_________ |
| 5 | መድኃኒት/ቶች መጠቀም ከጀመሩ ምን ያህል ጊዜ ሆነዎት? | በአመት____________, በወር__________________________ |
| 6 | የመድኃኒት ምላሽ ሰጪነት ሁኔታ | 1. Drug responsive 2. Drug resistant 3. Undefined |

| ***Steps 3 የፊዚካል ልኬቶች*** |
| --- |

| **የደም ግፍት** | | | |
| --- | --- | --- | --- |
| ***ጥያቄዎች*** | ***መልስ*** | | ***መለያ*** |
| የጠያቂው መለያ ቁጥር |  | └─┴─┘ | M1 |
| የደም ግፍት መለኪያ የሚውለው መሳሪያ መለያ ቁጥር |  | └─┴─┘ | M2 |
| የመለኪያው የካፍ መጠን (Cuff size) | Small | 1 | M3 |
|  | Medium | 2 |  |
|  | Large | 3 |  |
| የመጀመሪያ ንባብ | Systolic (mmHg) | └─┴─┴─┘ | M4a |
|  | Diastolic (mmHg) | └─┴─┴─┘ | M4b |
| ሁለተኛ ንባብ | Systolic (mmHg) | └─┴─┴─┘ | M5a |
|  | Diastolic (mmHg) | └─┴─┴─┘ | M5b |
| ሶስተኛው ንባብ | Systolic (mmHg) | └─┴─┴─┘ | M6a |
|  | Diastolic (mmHg) | └─┴─┴─┘ | M6b |
| ባለፋት ሁለት ሳምንት የደም ግፊትዎን ለመቆጣጠር በሐኪም መድሐኒት ታዞሎት እየወሰዱ ነው? | Yes | 1 | M7 |
|  | No | 2 |  |
| **ቁመት እና ክብደት** | | | |
| እርጉዝ ነዎት (ለሴቶች ብቻ) | Yes | 1 If *Yes, go to M 16 a-c* | M8 |
|  | No | 2 |  |
| የጠያቂው መለያ ቁጥር |  | └─┴─┘ | M9 |
| ለክብደት እና ቁመት መለኪያ የሚውለው መሳሪያ መለያ ቁጥር | Height | └─┴─┘ | M10a |
|  | Weight | └─┴─┘ | M10b |
| ቁመት | in Centimetres (cm) | └─┴─┴─┘. └─┘ | M11 |
| ክብደት  በጣም ወፍራም ሆነው ከሚዛኑ መጠን በላይ ከሆነ 666.00 ይጻፋ | in Kilograms (kg) | └─┴─┴─┘.└─┘ | M12 |
| *ወገብ* | | | |
| ወገብ ለመለካት የሚውለው መሳሪያ መለያ ቁጥር |  | └─┴─┘ | M13 |
| የወገብ ስፋት | in Centimetres (cm) | └─┴─┴─┘.└─┘ | M14 |

| **Step 4 የባዩኬሚካል ልኬቶችን በተመለከተ** |
| --- |

| *የደም የጉልኮስ በተመለከተ* | | | |
| --- | --- | --- | --- |
| ***ጥያቄዎች*** | ***መልስ*** | | ***መለያ*** |
| *ባለፋት 12 ሰአታት ከውሃ ውጪ የጠጣኸው ወይም የተመገብከው ነገር አለ* | Yes | 1 | B1 |
|  | No | 2 |  |
| *የጠያቂው መለያ ቁጥር* |  | └─┴─┘ | B2 |
| *የመለኪያው መለያ ቁጥር* |  | └─┴─┘ | B3 |
| *የደም ናሙና የተወሰደበት ሰአት (24 ሰአት ፎርማት)* | Hours: minutes | └─┴─┘: └─┴─┘  hrs mins | B4 |
| *ምግብ ሳይወሰድ የተለካ የደም የጉልኮስ መጠን mg/dl* | mg/dl | └─┴─┴─┘.└─┘ | B5 |
| *በዛሬው እለት ለስኳር ሕመምህ ኢንሱሊን ወይም ሌላ መድሃኒት ወስደሃል?* | Yes | 1 | B6 |
|  | No | 2 |  |
| **የኮሌስትሮል(የደም ውስጥ ስብ/ቅባት)** | | | |
| *የኮሌስትሮል መጠን( TC) mg/dl* | mg/dl | └─┴─┴─┘.└─┘ | B8 |
| *ባለፋት ሁለት ሳምንታት ከፍ ያለውን የኮሌስትሮል መጠን ለመቀነስ መድሃኒት ወስደዋል?* | Yes | 1 | B9 |
|  | No | 2 |  |
| *የሽንት ውስጥ የጨው እና የኘሮቲን መጠን* | | | |
| *የሽንት ናሙና ስወሰድ ከውሃ ውጪ የጠጣኸው ወይም የተመገብከው ነገር አለ?* | Yes | 1 | B10 |
|  | No | 2 |  |
| *የጠያቂው መለያ ቁጥር* |  | └─┴─┘ | B11 |
| *የሽንት ናሙና የተወሰደበት ሰአት (24 ሰአት ፎርማት)* | Hours: minutes | └─┴─┘: └─┴─┘  hrs mins | B13 |

| **ተራይግላይሰራይድ እና ከፍተኛ ደንሲቲ ያለዉ የኮሌስትሮል(የደም ውስጥ ስብ/ቅባት) (Triglycerides and HDL Cholesterol)** | | | |
| --- | --- | --- | --- |
| ***ጥያቄዎች*** | ***መልስ*** | | ***መለያ*** |
| *HDL-C* | mg/dl | └─┴─┴─┘.└─┘ | B17 |
| *TG* | (mg/dl) └─┴─┴─┘.└─┘ |  |  |
| LDL-C | mg/dl └─┴─┴─┘.└─┘ |  |  |

**እናመሰግናለን**
