# Supplementary material for: Metabolic syndrome and its associated factors among epileptic patients at Dessie Comprehensive Specialized Hospital, Northeast Ethiopia; a hospital-based comparative cross-sectional study
Source: PLoS One. 2022 Dec 29;17(12):e0279580. doi: 10.1371/journal.pone.0279580 (PMC9799290; doi:10.1371/journal.pone.0279580)
Supplement: Attachment — Submitted filename: Response to Reviewers.docx [file pone.0279580.s004.docx]

**Rebuttal letter**

Dear editors Dr. Rick J. Jansen, and dear reviewers,

We would like to thank you for giving us the opportunity to submit a revised version of our manuscript titled “*Metabolic syndrome and its associated factors among epileptic patients at Dessie Comprehensive Specialized Hospital, Northeast Ethiopia; a hospital-based comparative cross-sectional study”.* We are too grateful to you and the reviewers for taking the time and effort to read our manuscript and offer insightful comments. Your informed and helpful feedback helped us think of potential changes for the current version. We have cautiously considered all the comments and tried our best to address every one of them. We believe the manuscript has improved and hope the manuscript after careful revisions meet your high standards. The authors welcome further constructive comments if any.

Please see below for a point-by-point response to comments and concerns. Comments are shown in black, followed by our answer/responses in dark blue. All changes within the manuscript are highlighted blue.

Best wishes,

**Editor’s Comment,**

Dear Dr. kassaw,
Thank you for submitting your manuscript to PLOS ONE. After careful consideration, we feel that it has merit but does not fully meet PLOS ONE’s publication criteria as it currently stands. Therefore, we invite you to submit a revised version of the manuscript that addresses the points raised during the review process.
Please include the following items when submitting your revised manuscript:

- A rebuttal letter that responds to each point raised by the academic editor and reviewer(s). You should upload this letter as a separate file labeled 'Response to Reviewers'.
- A marked-up copy of your manuscript that highlights changes made to the original version. You should upload this as a separate file labeled 'Revised Manuscript with Track Changes'.
- An unmarked version of your revised paper without tracked changes. You should upload this as a separate file labeled 'Manuscript'.

If you would like to make changes to your financial disclosure, please include your updated statement in your cover letter. Guidelines for resubmitting your figure files are available below the reviewer comments at the end of this letter.

If applicable, we recommend that you deposit your laboratory protocols in [protocols.io](http://protocols.io/) to enhance the reproducibility of your results.

Journal requirements:

When submitting your revision, we need you to address these additional requirements.

1. Please ensure that your manuscript meets PLOS ONE's style requirements, including those for file naming. The PLOS ONE style templates can be found at

2. Thank you for stating the following financial disclosure:

“there has been no funding for this work that could have influenced its outcome”

At this time, please address the following queries:

a) Please clarify the sources of funding (financial or material support) for your study. List the grants or organizations that supported your study, including funding received from your institution.

b) State what role the funders took in the study. If the funders had no role in your study, please state: “The funders had no role in study design, data collection and analysis, decision to publish, or preparation of the manuscript.”

c) If any authors received a salary from any of your funders, please state which authors and which funders.

d) If you did not receive any funding for this study, please state: “The authors received no specific funding for this work.”

Please include your amended statements within your cover letter; we will change the online submission form on your behalf.

3. Thank you for stating the following in your Competing Interests section:

“No authors have competing interests”

4. In your Data Availability statement, you have not specified where the minimal data set underlying the results described in your manuscript can be found. PLOS defines a study's minimal data set as the underlying data used to reach the conclusions drawn in the manuscript and any additional data required to replicate the reported study findings in their entirety. All PLOS journals require that the minimal data set be made fully available.

Upon re-submitting your revised manuscript, please upload your study’s minimal underlying data set as either Supporting Information files or to a stable, public repository and include the relevant URLs, DOIs, or accession numbers within your revised cover letter.

5. PLOS requires an ORCID iD for the corresponding author in Editorial Manager on papers submitted after December 6th, 2016. Please ensure that you have an ORCID iD and that it is validated in Editorial Manager. To do this, go to ‘Update my Information’ (in the upper left-hand corner of the main menu), and click on the Fetch/Validate link next to the ORCID field. This will take you to the ORCID site and allow you to create a new iD or authenticate a pre-existing iD in Editorial Manager.

6. Your ethics statement should only appear in the Methods section of your manuscript. If your ethics statement is written in any section besides the Methods, please delete it from any other section.
**Additional Editor Comments:**
Please make sure to address all reviewer comments fully with specific attention to the comment on reproduction of existing work. Thank you!

**Response to the editor:** We thank you again for the insightful comments given to our manuscript which obviously further improves the quality of our work. We have been able to incorporate changes to reflect all the comments and suggestions provided.

Being specific on the points you raised:

#1. We will deposit soon our laboratory protocols in **protocols.io.**, as it has been recommended

#2. Regarding financial disclosure, we have not made a change (we didn’t receive any funding for this study). Hence, we have stated “The authors received no specific funding for this work” on the cover letter.

#3. On the competing interest section, we have completed the Competing Interests on the online submission form to state any Competing Interests and have stated on the cover letter as “The authors have declared that no competing interests exist”.

#4. In Data Availability statement, we have specified the minimal data set underlying the results described in our manuscript (as Supporting information files).

#5. ORCID iD for the corresponding author has been linked to Editorial Manager profile.

#6. We incorporate the ethics statement in the methods section of our manuscript.

#7. We have fully addressed all the reviewer comments and suggestions.

**Comments from reviewers and point – to – point response**

**Reviewer #1:**

**Comments**:  in methodology part the selection of apparently healthy individuals is not clear. so, it needs revision the selection of apparently healthy individual criteria must be written on the material and method part.

**Response to the comments:** Thanks for your kind reminders. We found your comment helpful and has now been written clearly as suggested. An equal number of age and sex-matched apparently healthy volunteer subjects (apparently healthy care givers/ patient attendants who full fill the inclusion criteria) were enrolled in the comparison group. The participants were selected consecutively. [ Page-6, L127-129], on unmarked version of our revised manuscript.

**Reviewer #2:**

**Comment 1**[Abstract]: page 2 line 33, 36 & 39: the association of BMI with MS needs to be described clearly (higher vs lower BMI).

**Response: thank you very much for your suggestion.** Although we agree that categorizing BMI as high or low is good to assess its association with MS, continuous independent variables can also be fitted as such and would have high statical power in predicting association, strong prediction contribution to the outcome variable (Jill C. Stoltzfus, 2011). Accordingly, we take BMI as continuous variable and asses its association with MS. The clarified result has presented on “Associated factors of metabolic syndrome among the epileptic groups” section [ Page-20, L341-346, or on Table 7(Page 21) and Table 8 Page 22)].

**Comment 2[**Introduction]: paragraph one and two can be merged and re-written considering the paper is meant for the scientific community with an understanding of what epilepsy and metabolic syndrome are. The emphasis in the introduction should be on the cross-link between epilepsy and metabolic syndrome.
**Response**: Thanks for your kind reminders. We found your comment is helpful and have revised accordingly.

**Comment 3 [**Study participants: Line 110-121]: The authors would benefit in adding statement explaining why they chose to use healthy controls.

**Response:** We thanks for the suggestion and incorporated it in our revised manuscript [Page-5, L107-109]. To be clearer, we initially intended to show “whether MS is a problem in epileptic population in the study setting, or to assess whether MS is associated with epilepsy or weather epilepsy and related issues are a risk for MS”. Accordingly, we tried to compare the presence of MS between epileptic and non-epileptic study participants even though the small sample size we enrolled and the study design we used may not sufficiently ascertain. To minimize the effect of confounder, we chose non-epileptic group from healthy individuals.

**Comment 4 [Result and discussion]:** why did the author prefer to use both the IDF and NCEP-ATP III criteria in the description of the result and discussion? Which one is used in the study setting? The authors should use one of the criteria and present the other as a sensitivity analysis by providing the result as a supplementary file.

**Response:** we are grateful for the comment! Using a single criterion may over estimate or under estimate the magnitude of MS. Hence, we used both the IDF and NCEP-ATP III criteria independently in our setting. Moreover, we did sensitivity analysis and provided the result as a supplementary file (S1).

**Comment 5 [Discussion]:** The authors frequently used statements mentioned in the result part in their discussion and focused on comparing their result with others. Instead of comparing the finding of your study with other studies, the focus should be on explaining the consequence of the study finding on the study population.
**Response to comment:**  We thank you for you’re a very interesting comment. As the Purpose of discussion is to describe, analyzes and interpret/justify findings in relation to the existing literature, theory and practice ((Skelton.J. et al, 2000), (Shona Mc Combes, 2022), (Oner Şanli, et al, 2013)) we were trying to do accordingly. We believe the “consequence of the study finding on the study population” is addressed in a conclusion section [Page-29, Line no. 512-518].

**Comment 6 [Strength and Limitation of the Study]:** Line 501 “It also includes healthy controls as a comparative group and treatment-naive epileptic patients.” …. did the study include treatment naïve epileptic patients as a control group. As this is not reflected in neither the methods nor result section.

**Response to comment:** Thanks for your kind question. We rather considered current treatment status of epileptic participants as a variable and fitted into logistic regression analysis (Current anti-epileptic drugs (AEDs) use: On mono therapy, On Poly therapy, Not on antiepileptic-agents/ treatment naïve), and tried to show weather the MS is because of the epilepsy by itself or because of the different anti-epileptic drugs [Table 7(Page 21) and Table 8 Page 22)].

**Reviewer #3:**

**Comment 1:** I have reviewed with great interest the original research article entitled "Metabolic syndrome and its associated factors among epileptic patients at Dessie Comprehensive Specialized Hospital, Northeast Ethiopia; a hospital-based comparative cross-sectional study" which was intended to assess the magnitude of metabolic syndrome among epileptic patients. However, I have the following concerns: A sample size of 102 may not be enough to say the prevalence/magnitude of metabolic syndrome among epileptic patients; rather, it might provide evidence for the presence of metabolic syndrome in epileptic patients in comparison with another 102 healthy controls simply. However, whether the metabolic syndrome is because of the anti-epileptic drugs or due to the epilepsy disease per se should be clearly discussed in this manuscript.

**Response to the comment:** We are very appreciative for your insightful suggestions and comments. We have gone through your comments carefully and tried our best to address them. Even though the study design we choice (cross-sectional study design) cannot sufficiently ascertain whether the metabolic syndrome is because of the anti-epileptic drugs or due to the epilepsy disease per se (which has been stated as a limitation), the likelihood of MS, given its criteria, has been adjusted for possible confounding variables using a multivariate logistic regression model [Table 7(Page 21) and Table 8 (Page 22)]. Hence, Current anti-epileptic drugs (AEDs) use status (On mono therapy, On Poly therapy, Not on antiepileptic-agents/ treatment naïve) has been adjusted to….

**Comment 2**: Using the nonstandard abbreviations in the abstract section of the manuscript is not
recommended. However, the authors used several abbreviations in the abstract section, and the
author should write the long form of abbreviations for the first time, e.g., DCSH, MS, AEDs.

**Response to the comment**: thank you for pointing this out. We agree with this comment and has now been amended in the revised abstract.

**Comment 3:** Since they did not have a list of healthy controls, how could they use a systematic random sampling technique to select patient attendants or caregivers? The author said that they used systematic random sampling techniques to select the control groups, which is impractical without having the frame.

**Response to the comment**: We thanks for a very interesting doubt you raised. Honestly speaking, volunteer healthy patient attendants were selected consecutively and we are very sorry for a typing fault that has been done in editing of the manuscript. We have now added the corrected content to the manuscript on [Page-6, Line no. 127-129].

**Comment 4**: The sampling technique and procedures are not well explained for cases and control groups separately. They should review the findings in relation to the sampling method.

**Response to the comment**: Thank you for the comments. We have revised these points according to the suggestions.
