# Supplementary material for: Metabolic syndrome and its associated factors among epileptic patients at Dessie Comprehensive Specialized Hospital, Northeast Ethiopia; a hospital-based comparative cross-sectional study
Source: PLoS One. 2022 Dec 29;17(12):e0279580. doi: 10.1371/journal.pone.0279580 (PMC9799290; doi:10.1371/journal.pone.0279580)
Supplement: Attachment — Submitted filename: Response to Reviewers.docx [file pone.0279580.s005.docx]

**Rebuttal letter**

Dear editors Dr. Rick J. Jansen,

We would like to give our deepest gratitude again for your comments, support, and invitation for resubmitting our manuscript, entitled “*Metabolic syndrome and its associated factors among epileptic patients at Dessie Comprehensive Specialized Hospital, Northeast Ethiopia; a hospital-based comparative cross-sectional study”**, t*oo. Your informed and helpful feedback helped us re-think potential changes for the current version. We have considered all the comments and tried our best to address every one of them. We now believe the manuscript has improved to meet your high standards. The authors welcome further constructive comments if any.

Please see below for our response to the comments. Comments are shown in black, followed by our responses in blue. All changes made to the original version of the manuscript are highlighted in blue, with in the marked-up copy of our manuscript.

Best wishes,

**Editor’s Comment,**

**Comment 1 [Abstract]:** Please make sure to remove all nonstandard abbreviations from the abstract. Also, please address the comment from the reviewer suggesting this is a mostly reproduced manuscript of a published manuscript. Thank you

**Response to the comments:** Thanks for your kind reminders. We found your comment helpful and has now been addressed clearly as suggested.

**Comment 2** [**Journal Requirements]:** Please review your reference list to ensure that it is complete and correct. If you have cited papers that have been retracted, please include the rationale for doing so in the manuscript text, or remove these references and replace them with relevant current references. Any changes to the reference list should be mentioned in the rebuttal letter that accompanies your revised manuscript. If you need to cite a retracted article, indicate the article’s retracted status in the References list and also include a citation and full reference for the retraction notice.

**Response**: we are grateful for the comments and suggestions. Up on revising our references’ completeness and correctness, we noticed that two retracted papers had been cited (article “6” and “21” on original reference list), but now we remove and replace them with the current reference, as suggested.

**Additional Editor Comments:**

Please make sure to remove all nonstandard abbreviations from the abstract. Also, please address the comment from the reviewer suggesting this is a mostly reproduced manuscript of a published manuscript. Thank you

**Responses:** We are very appreciative of your kind reminders again which further improve the quality of our work. We have tried to address previous comments and suggestions from reviewers’ side, even though we couldn’t find their additional comments in this round.
